# Principles and Practical Steps of Simplifying the Construction of the Cushion Curves of Closed-Cell Foam Materials

**DOI:** 10.3390/polym17172292

**Published:** 2025-08-24

**Authors:** Deqiang Sun, Pengcheng Qiu, Hongjuan Chen, Xinyuan Zhang, Siyu Wang

**Affiliations:** 1 College of Bioresources Chemical and Materials Engineering, Shaanxi University of Science and Technology, Xi’an 710021, China; qufuence@163.com (P.Q.); zhangsudhh112@163.com (X.Z.); 2 College of Art and Design, Shaanxi University of Science and Technology, Xi’an 710021, China; chenhongjuan@sust.edu.cn; 3 Shaanxi Provincial Institute of Product Quality Supervision and Inspection, Xi’an 710061, China; kindle2llxxsh@163.com

**Keywords:** closed-cell foam materials, categories of cushion curves, construction principles of cushion curves, simplified construction methods of cushion curves, dynamic cushion factor-dynamic stress method, practical steps of constructing cushion curves

## Abstract

The cushion curves of cushioning materials play crucial roles in scientific and reliable cushioning designs and in reducing damage losses for fragile products during distributions. The construction methods of cushion curves of closed-cell foam materials (CFMs) mainly include the Janssen factor, Rusch curve, cushion factor, and energy absorption diagram. The construction principle of these methods is reviewed in detail, and their disadvantages are mainly discussed. According to relevant ASTM and GB/T experimental standards, the peak acceleration–static stress cushion curve is based on dynamic impacts, which are most consistent with the dropping situation of product packages, so this kind of cushion curve is the standard and most widely applied for product cushioning designs. However, when generating the peak acceleration–static stress cushion curves, the experimental work is extremely huge. Three methods, namely the dynamic factor method, dynamic stress–dynamic energy method, and dynamic cushion factor–dynamic energy method, can significantly reduce the experimental workload and simplify constructing cushion curves. The novel dynamic cushion factor–dynamic stress method is proposed to simplify constructing the cushion curves. The practical generation steps of constructing cushion curves based on the four simplified methods are created and presented in detail.

## 1. Introduction

During the transportation of products, due to poor packaging, the economic loss of products caused by falling, impact, and vibration is significant [1]. By adding cushioning pads with appropriate thickness and load-bearing area between the internal products and the outer packaging box, the products can be well-protected [2]. Common cushioning materials are mostly cellular materials, such as open- and closed-cell foams, honeycomb paperboard, corrugated paperboard, and so on. These materials have good cushioning properties, because they can undergo large deformations while the force acting on the product does not change significantly or even remains almost constant before absorbing the total kinetic energy of product under dynamic loads [3]. In addition, these cellular materials are lightweight, waterproof, and soft, and also have excellent performance of resilience, energy absorption, cushioning and vibration damping, sound and heat insulation, corrosion resistance, weather and bacteria resistance, and ease molding [3]. Therefore, they are widely used as cushioning and protective materials in construction, aerospace, transportation, furniture, military, product packaging, and other fields [3]. Unlike the open-cell foam materials, the CFMs, owing to the air entrapped within their closed cells, are able to rebound after undergoing significant deformation and absorbing substantial dynamic energy [3], thereby exhibiting good cushioning performance. In the field of cushioning protection, the CFMs are typical cushioning protective materials that have been in existence and widely used for a long time [4].

To carry out reliable cushioning designs of CFMs, firstly, it is necessary to construct their cushion curves so that the appropriate thickness and load-bearing area of cushioning pads can be determined based on the information such as the fragility of the protected object (product), drop height, and so on [5,6]. Reviewing the development history of cushion curves for CFMs, there are some construction methods [7], such as the Janssen factor [8], Rusch curve [9], cushion factor [2], and energy absorption diagram [10]. Based on the Janssen factor method, a cushion curve of the Janssen factor with respect to energy absorption per unit volume can be depicted [8]. Employing the Rusch curve method, the cushion curve of normalized impact peak stress versus normalized per unit volume impact energy can be drawn [9]. Using the cushion factor method, the cushion curves of peak acceleration with respect to static stress [11] and cushion factor versus stress [12] can be constructed. Based on the energy absorption diagram method, the cushion curves of normalized energy absorption per unit volume versus stress [3] as well as normalized stress versus relative density [13] can be developed for the CFMs with the same base material under a certain strain rate (or temperature). For the CFMs, in the plateau phases of their stress–strain curves, the stress is strengthened with the increase in strain, which results in their specific energy absorption diagrams [3], due to the contribution of air pressure inside their closed cells, unlike the open-cell foam materials. Many researchers [13,14,15] have specifically pointed out that the energy absorption diagram method is the best way, while many others [4,16,17,18] did not use this method when evaluating the cushioning performance of CFMs. Therefore, it is necessary to review the construction principles of these cushion curves and clarify their advantages and disadvantages, in order to indicate the optimal rationality of cushion curves constructed by using the cushion factor method and its consistency with actual dropping situations of packages.

However, when using the cushion factor method to construct the peak acceleration-static stress cushion curves according to relevant experimental standards, such as ASTM d1596-2014 [11] and GB/T 8167-2008 [19], a large number of impact tests should be carried out for the same cushioning pads with various thicknesses, under different drop heights [7,12,14,19,20,21], resulting in an unusually large amount of test workload. For the CFMs, under dynamic impact loadings, certain relationship expressions can be established between their static stress, dynamic stress, dynamic energy, or dynamic cushioning coefficient. Thereupon, to simplify the construction process of the peak acceleration–static stress cushion curves based on a finite number of impact tests of CFMs, some methods appeared, such as dynamic factor method [22,23,24], dynamic stress–dynamic energy method [25,26,27,28], dynamic cushion factor–dynamic energy method [2,29], and so on. Inspired by these methods, a novel simplified construction method—the dynamic cushion factor–dynamic stress method is proposed for the CFMs. Corresponding to the construction principles of these simplified methods, the practical and detailed generation steps of cushion curves are put forward and established.

## 2. Categories and Construction Principles of Cushion Curves

### 2.1. Janssen Factor

Woolam [8] first applied the Janssen factor method to characterize the dynamic cushioning performance of materials based on the response data of cushioning pads, measured in the drop hammer impact tests. The drop hammer with some mass blocks impacting the cushioning pad (specimen) is equivalent to the protected object. The total mass of the drop hammer and its mass blocks is assumed to be *m*, and its impact velocity when contacting the specimen is called the initial velocity *v*. Assuming that there is an ideal cushioning pad, which absorbs energy under a constant acceleration of *a*_i_, the *a*_i_ value can be calculated through equating the impact energy to the work performed by the constant force on the ideal cushioning pad with the displacement equal to the initial thickness of cushioning pad in impact direction *t*, as

(1) $$mv^{2}/2=ma_{i}t$$

So

(2) $$a_{i}=\frac{v^{2}}{2t}$$

To describe the acceleration efficiency of absorbing the kinetic energy for the CFM, the ratio of actual impact peak acceleration *a*_p_ to *a*_i_, viz., the Janssen factor *J*, is

(3) $$J=\frac{a_{p}}{a_{i}}$$

**Figure 1:**
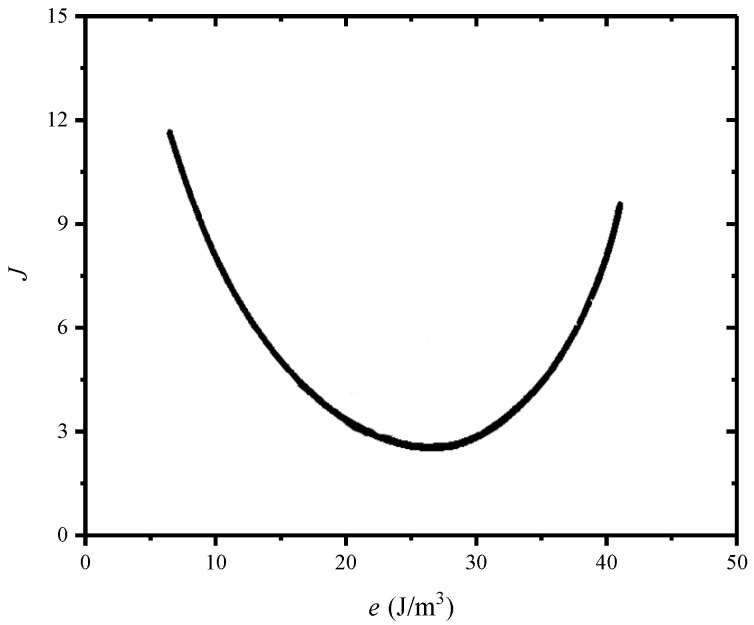
A typical *J*–*e* curve of CFM plotted according to the method in the reference [8].

The *J* factor depends on the impact energy, and its value is very high at low and high impact energy and reaches a minimum at a moderate impact energy, under a given v/t ratio. For the cushioning pad of some CFM with the fixed *t*, different values of impact energy can be applied by changing the mass blocks installed on the drop hammer, under a certain drop height *h*. Under some given v/t ratio, the typical cushion curve based on the *J* factor is shown in Figure 1, where the abscissa is the energy absorption per unit volume (an energy density with the units of J/m^3^, N/m^2^, or Pa) of the cushioning pad, *e*. Assuming that the bearing area of the cushioning pad in the loading direction is *A*, then

(4) $$e=\frac{mv^{2}}{2At}$$

For the ideal cushioning pads, the *J* factor would be in unity with values of about two. Although the *J*–*e* cushion curves can be used to compare the acceleration efficiencies of the energy absorption of different CFMs, they fail to connect the energy absorption with the deformation mechanism of the CFM and are only an empirical measure. Moreover, to construct the *J*–*e* cushion curves for one kind of CFM with different densities, a large number of tested data of the cushioning pads with different thicknesses needs to be collected under different drop heights.

### 2.2. Rusch Curve

Rusch [9] improved the Janssen factor method still based on the measured data in drop hammer impact tests of CFMs. He noticed that the stress–strain curve of CFM under quasi-static compression can be determined by the empirical analytical function *ψ*(*ε*), viz.,

(5) $$\sigma =E^{*}\psi \left(\varepsilon \right)\varepsilon$$

where *σ* is the compressive stress on cushioning pad, *ε* is the corresponding strain, and E* is the static elastic modulus of CFM. According to the experimental experience, *ψ*(*ε*) is expressed as

(6) $$\psi \left(\varepsilon \right)=w\varepsilon^{-n}+r\varepsilon^{s}$$

where *w*, *n*, *r*, and *s* are fitting constants of the *σ*–*ε* curve of CFM. The above two formulas determine the *σ*–*ε* curve of CFM. The impact energy absorption efficiency *K*, as the reciprocal of *J* factor, can be calculated from Expressions (2) and (3) as

(7) $$K=\frac{v^{2}}{2ta_{p}}$$

Rusch [9] also defined a dimensionless coefficient *I*, which is the energy absorption per unit volume normalized by the static elastic modulus of CFM, expressed as

(8) $$I=e/E^{*}$$

From Equations (4), (7), and (8), it is obtained that

(9) $$\frac{I}{K}=\frac{mv^{2}}{2AtE^{*}}\cdot \frac{2ta_{p}}{v^{2}}=\frac{ma_{p}}{AE^{*}}=\frac{\sigma_{m}}{E^{*}}$$

where *σ*_m_ is the impact peak stress of the drop hammer.

**Figure 2:**
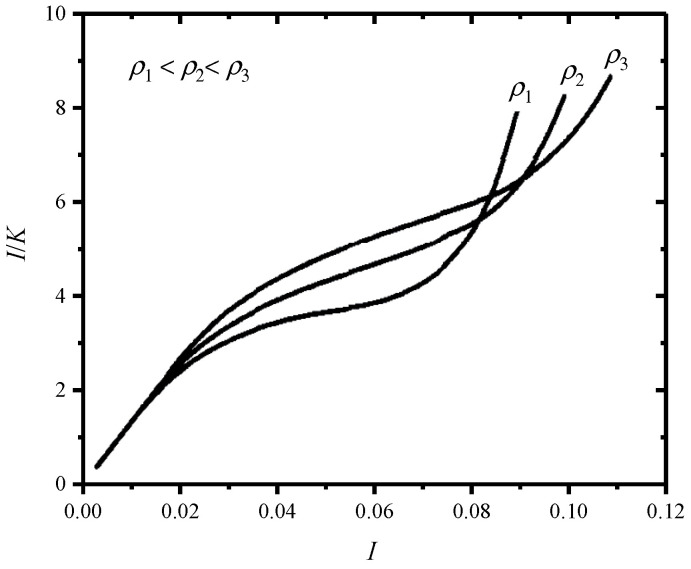
A typical *I*/*K*–*I* curve of CFM plotted according to the method in the reference [9].

It can be seen that the ratio of *I*/*K* is actually the impact peak stress normalized by the static elastic modulus of CFM. Therefore, the most proper CFM that absorbs a given amount of impact energy under the maximum allowable peak stress can be determined by the constructed *I*/*K*–*I* cushion curves. The typical *I*/*K*–*I* curves of some kind of CFMs with three different densities *ρ*_1_, *ρ*_2_, and *ρ*_3_ (*ρ*_1_ < *ρ*_2_ < *ρ*_3_) are shown in Figure 2. Both *I* and *K* can be linked to the *σ*–*ε* curve of CFM through the analytical function *ψ*(*ε*). The Rusch curve method is more general than the Janssen factor method, but it depends on the empirical function describing the shape of *σ*–*ε* curve and lacks sufficient physical and mechanical principles.

### 2.3. Cushion Factor

**Figure 3:**
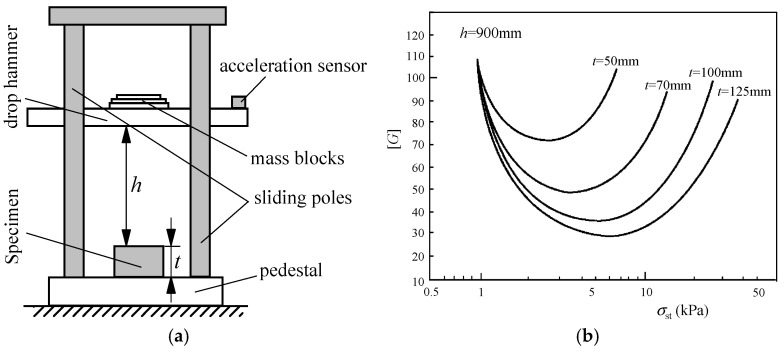
Principle of dynamic impact tests and cushion curves: (**a**) the schematic diagram of drop test apparatus; (**b**) typical [*G*]–*σ*_st_ curves reproduced from the reference [6].

Ge [2] systematically summarized the theoretical background of the cushion curve based on the cushion factor. The cushion curves based on the cushion factor are also obtained from the dynamic impact tests of cushioning pads, as shown in Figure 3.

#### 2.3.1. Cushioning Theory

The static stress *σ*_st_, generated by the total gravity of the drop hammer and its mass blocks placed on the cushioning pad, meets

(10) $$\sigma_{\mathrm{st}}=mg/A$$

where *g* is the acceleration of gravity. Assuming that the deformation of the cushioning pad in the impact direction is *x*, the corresponding transient reaction force of the cushioning pad to resist the impact load of the drop hammer is *F*(*x*), and the corresponding strain *ε* = *x*/*t*, with the strain rate $\dot{\varepsilon }=\dot{x}/t$. The acceleration of the drop hammer is assumed as *a*, and then, according to Newton’s second law, there is a relationship as

(11) $$F\left(x\right)=m\left(a+g\right)$$

The stress generated by the cushioning pad resisting the external impact *σ*(*ε*,$\dot{\varepsilon }$), which is related to *ε* and $\dot{\varepsilon }$, defined as

(12) $$\sigma \left(\varepsilon ,\dot{\varepsilon }\right)=F\left(x\right)/A$$

Then, the corresponding energy absorption per unit volume of cushioning pad *e*, is

(13) $$e=\int_{0}^{\varepsilon }\sigma \left(\varepsilon ,\dot{\varepsilon }\right)d\varepsilon$$

Because the initial velocity of the drop hammer when contacting the cushioning pad is known to be *v*, the equivalent drop height $h=v^{2}/2g$. When *x* comes up to the maximum deformation *x*_m_, the kinetic energy of the drop hammer is totally absorbed by the cushioning pad. Because *x*_m_ << *h* in general, then

(14) $$tA\int_{0}^{\varepsilon_{m}}\sigma \left(\varepsilon ,\dot{\varepsilon }\right)d\varepsilon =mg\left(h+x_{m}\right)\approx mgh=\frac{mv^{2}}{2}$$

where *ε*_m_ is the maximum strain of the cushioning pad corresponding to *x*_m_, and *ε*_m_ = *x*_m_/*t*. Thus, corresponding to *ε*_m_, the maximum energy absorption per unit volume of cushioning pad *e*_m_, is

(15) $$e_{m}=\int_{0}^{\varepsilon_{m}}\sigma \left(\varepsilon ,\dot{\varepsilon }\right)d\varepsilon =\frac{mgh}{At}=\sigma_{\mathrm{st}}\frac{h}{t}$$

The above expression can be transformed into

(16) $$\frac{1}{\sigma_{\mathrm{st}}}=\frac{h}{e_{m}t}$$

When *x*∈ (0, *x*_m_] and *ε* ∈ (0, *ε*_m_], *σ*_m_ refers to the maximum value of *σ* (*ε*,$\dot{\varepsilon }$). In combination with Equations (11) and (12), there must be an equation of

(17) $$\sigma_{m}=\frac{mg\left[G\right]+mg}{A}=\sigma_{\mathrm{st}}\left(\left[G\right]+1\right)$$

where [*G*] represents the fragility of the protected object, which also refers to the ratio of the peak acceleration of drop hammer *a*_p_ to *g*. From the above two formulas, it can be derived that

(18) $$\left[G\right]=\frac{\sigma_{m}}{e_{m}}\cdot \frac{h}{t}-1$$

The cushion factor *C* is defined as

(19) $$C=\sigma_{m}/e_{m}$$

According to the above two equations, *C* can also be expressed as

(20) $$C=\frac{\left[G\right]+1}{h/t}$$

#### 2.3.2. Peak Acceleration–Static Stress Curve

The drop hammer impact test reproduces the dropping process of the product package, which has been widely used in the field of cushioning protection. According to ASTM d1596-2014 [11] and GB/T 8167-2008 [19], the [*G*]–*σ*_st_ curve is constructed based on such drop impact tests, which is the most reasonable and applicable cushion curve consistent with the actual dropping process of the package. The hammer impact testing machine consists of an impact test apparatus (schematically shown in Figure 3a), a testing machine controller, and data acquisition and processing system, and the last includes a charge amplifier, data acquisition card, data acquisition and processing software, and computer [30].

According to the above two test standards [11,19], the drop impact tests should be carried out for the cushioning pads of CFMs with a certain base material, density, and thickness, under a certain drop height. The cross-section of the specimen along the impact direction is generally rectangular, with the length and width not smaller than 4 inches. During the drop hammer impact process, the bottom surface of the specimen is fixed in the central region of the upper surface of the rigid support pedestal, and its upper bearing surface is parallel to the bottom surface of the drop hammer, to ensure that its center is in the same vertical line with the gravity center of the drop hammer. After falling freely from the designated height, the drop hammer impacts the specimen, with an acceleration sensor installed. After sampling and filtering by the data acquisition and processing system, the acceleration–time curve of the drop hammer can be obtained, so as to determine the corresponding maximum acceleration. The same specimen is impacted five times continuously, with a time interval of 1–30 min between two adjacent impacts. Generally, the ratio of the average value of the last four maximum accelerations during these five consecutive impacts, to *g*, is taken as the [*G*] value. The total mass of the drop hammer and its mass blocks divided by the cross-sectional area of the specimen along the impact direction is the corresponding *σ*_st_ value. By changing the magnitude of mass blocks attached to the drop hammer, the impact energy of the drop hammer can be adjusted to obtain different [*G*] and *σ*_st_ values. To generate one cushion curve, at least five different mass block combinations are required. In this way, a series of tested value points of (*σ*_st_, [*G*]) are obtained for one kind of cushioning pad with a thickness, under a certain drop height, and the final [*G*]–*σ*_st_ curve can be constructed by means of curve fitting.

For the cushioning pads with different thicknesses, the above drop test process is repeated under different drop heights, and a series of [*G*]–*σ*_st_ cushion curves can be plotted under different combinations of *h* and *t*.

#### 2.3.3. Cushion Factor–Stress Curve

The CFM is generally sensitive to $\dot{\varepsilon }$ (temperature *T*) [20]. If the effect of strain rate can be neglected, the *σ*–*ε* curve of dynamic impact can be replaced by the corresponding one of quasi-static compression [31], obtained through the experimental method according to the test standard GB/T 8168–2008 [12]. If the effect of strain rate is considered, in order to improve the approximation of *σ*–*ε* curves between the compression and impact tests, the dynamic compression experiment can be carried out under the strain rate of ${\dot{\varepsilon }}_{0}=v/t$. Assuming that the reaction force and displacement of the cushioning pad to the upper compressive plate are *F* and *u*, respectively, under dynamic compression, the corresponding stress and strain, *σ* and *ε*, are

(21) $$\sigma \left(\varepsilon ,{\dot{\varepsilon }}_{0}\right)=\frac{F}{A},\;\varepsilon =\frac{u}{t}$$

Hereby, the *C*–*σ* cushion curve can be drawn according to the test standard GB/T 8166-2011 [6]. Integrating the *σ*–*ε* curve, the corresponding energy absorption per unit volume of cushioning pad *e*_0_, can be obtained according to the following formula of

(22) $$e_{0}=\int_{0}^{\varepsilon }\sigma \left(\varepsilon ,{\dot{\varepsilon }}_{0}\right)d\varepsilon$$

At this time, the cushion factor refers to

(23) $$C=\sigma \left(\varepsilon ,{\dot{\varepsilon }}_{0}\right)/e_{0}=\sigma \left(\varepsilon ,{\dot{\varepsilon }}_{0}\right)/\int_{0}^{\varepsilon }\sigma \left(\varepsilon ,{\dot{\varepsilon }}_{0}\right)d\varepsilon$$

Miltz and Gruenbaum [32] mentioned the concept of energy absorption efficiency *E*_e_, which is the reciprocal of *C*, expressed as

(24) $$E_{e}=\int_{0}^{\varepsilon }\sigma \left(\varepsilon ,{\dot{\varepsilon }}_{0}\right)d\varepsilon /\sigma \left(\varepsilon ,{\dot{\varepsilon }}_{0}\right)$$

**Figure 4:**
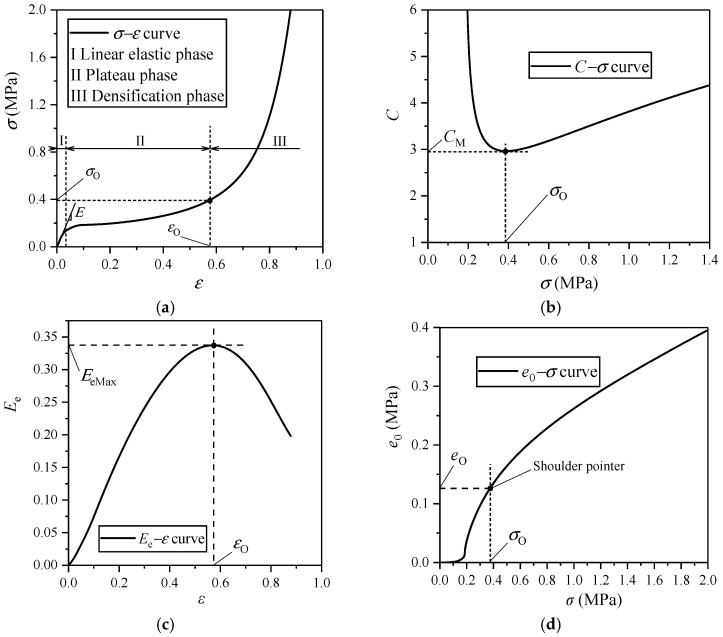
Typical response curves of some EVA CFM under compression plotted according to the method in the reference [35]: (**a**) *σ*–*ε* curve; (**b**) *C*–*σ* curve; (**c**) *E*_e_–*σ* curve; (**d**) *e*_0_–*σ* curve.

Chen et al. [19] used the mechanical indicators of *e*, *C*, and *E*_e_ to describe the comprehensive behaviors of ethylene-vinyl acetate (EVA) CFMs [33,34] under quasi-static and dynamic compressions in detail. The typical *σ*–*ε* curve of EVA CFM includes three deformation stages: linear elastic phase I, plateau phase II, and densification phase III (Figure 4a). The stress–strain relationship is approximately linear in phase I where the slope of the straight-line segment is the elastic modulus of CFM *E*, under this strain rate (Figure 4a); then, the foam yields with some cell walls in foam buckled, following by the non-linear long plateau stress in phase II; due to the contribution of air pressure inside the foam cells, the stress is strengthened with the increase in strain, which produces a stress plateau (Figure 4a). For the CFM, when the stress reaches a certain degree, *C* has a minimum value called the minimum cushioning coefficient *C*_Min_ (Figure 4b); the corresponding energy absorption efficiency is a peak value that is called the maximum energy absorption efficiency *E*_eMax_ (Figure 4c), which means that its energy absorption capacity reaches the best; therefore, the corresponding strain, stress, and energy absorption per unit volume are called the best strain *ε*_O_, the best stress *σ*_O_, and the best energy absorption per unit volume *e*_O_ (Figure 4a,d), respectively; accordingly, the shoulder point appears on the *e*–*σ* curve (Figure 4d).

*ε*_O_ is approximately equal to densification strain, and its calculation formula under static compression is [3,36]

(25) $$\varepsilon_{O}=1-1.4\frac{\rho }{\rho_{s}}=1-1.4\rho^{*}$$

where ρ* is the relative density of CFM, and *ρ*_s_ is the density of base material in CFM.

The cushioning process is the one in which the impact velocity first decreases, reverses, and then increases. If the *C*–*σ* curve of CFM corresponding to ${\dot{\varepsilon }}_{0}=v/t$ is used for cushioning design because the compressive speed in the whole compression process remains unchanged, it is bound to overestimate the cushioning performance of materials, resulting in poor packaging. On the other hand, if the cushioning design is carried out using the *C*–*σ* curve of quasi-static compression, the enhancement of the cushioning performance due to the dynamic effect of materials will be ignored, which will lead to over-packaging. When using the *C*–*σ* and [*G*]–*σ*_st_ curves to carry out the cushioning designs, the maximum stress is determined by the fragility of the protected object, corresponding to the CFM with the lowest cushion factor value [6]. In order to optimize the CFMs and their density, it is also necessary to carry out a large number of experiments on the different CFMs with various densities and thicknesses, even under different drop heights and compressive strain rates (or temperatures), which becomes complex.

### 2.4. Energy Absorption Diagrams

Maiti et al. [10] proposed the energy absorption diagram, which is an empirical method combined with physical models. It is general and attractive in the selection of CFMs. Gibson and Ashby [3] constructed the energy absorption models of some cellular materials and introduced how to optimize the density and thickness of CFMs by using the energy absorption diagrams.

**Figure 5:**
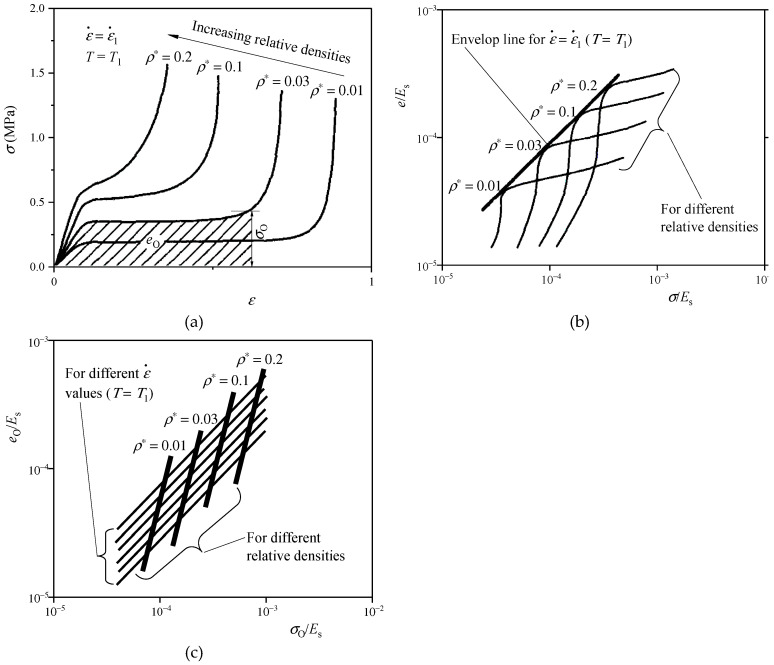
Construction principle of energy-absorption diagrams of CFMs plotted according to the method in the reference [37]: (**a**) *σ*–*ε* curves of CFMs with different ρ* values when $\dot{\varepsilon }={\dot{\varepsilon }}_{1}$ (*T* = *T*_1_); (**b**) envelop line when $\dot{\varepsilon }={\dot{\varepsilon }}_{1}$ (*T* = *T*_1_); (**c**) envelop lines for different $\dot{\varepsilon }$ values (*T* = *T*_1_).

The energy absorption diagram is generated by the *σ*–*ε* curves of CFM under compressions, considering the effect of strain rate (or temperature), as shown in Figure 5. Figure 5a depicts the *σ*–*ε* curves of a certain kind of CFM with different densities at a certain strain rate of $\dot{\varepsilon }={\dot{\varepsilon }}_{1}$(*T* = *T*_1_). For a given protected object, the best CFM is the one whose optimal stress value is just equal to the maximum allowable stress calculated from its fragility. It can be assumed that there is the CFM with the optimal relative density (ρ* = 0.03) that just absorbs the dynamic energy of the protected object without damaging it, under a certain peak stress level of *σ*_O_ and energy absorption *e*_O_; the same kind of CFMs with relative densities higher or lower than this value (ρ* = 0.03) produce higher stresses when totally absorbing the energy *e*_O_, causing the damage of protected object.

According to the curve generation method from Figure 4a–d, the corresponding *e*–*σ* curve is drawn for each *σ*–*ε* curve in Figure 5a, and then normalized by the elastic modulus of the base material of CFM *E*_s_, to obtain the corresponding *e*/*E*_s_–*σ*/*E*_s_ curve (Figure 5b). Likewise, there is one shoulder point on each *e*/*E*_s_–*σ*/*E*_s_ curve, and all shoulder points can be approximately located in a straight line passing through the coordinate origin (the bold line in Figure 5b) [35], which is called the envelope line passing through these shoulder points, describing the relationship between *e*_O_ and *σ*_O_ of the same kind of CFMs with different densities, when loaded at a certain strain rate of $\dot{\varepsilon }={\dot{\varepsilon }}_{1}$(*T* = *T*_1_).

Viewing *e*_O_ and *σ*_O_ as variables, in the same coordinate system, the energy absorption diagram of the same kind of CFMs with different densities can be depicted by connecting the points of (*e*_O_, *σ*_O_) at different strain rates with lines (Figure 5c). The thin solid line in Figure 5c is the envelope line through the shoulder points on the *e*/*E*_s_–*σ*/*E*_s_ curves of the same kind of CFMs with different densities under a certain strain rate (or temperature); The thick solid line is the line connecting the shoulder points on the *e*/*E*_s_–*σ*/*E*_s_ curves of the same kind of CFMs with the fixed relative density under different strain rates (or temperatures); For the same kind of CFMs with different relative densities, each of these thick and thin solid lines approximately forms a straight line [3].

Gibson and Ashby [3] gave the physical models of energy absorption diagrams for the CFMs. However, the actually depicted *e*/*E*_s_–*σ*/*E*_s_ curves of CFMs often have wide shoulders without distinct shoulder points. It is often very difficult to accurately determine the contact point between the envelope line and each *e*/*E*_s_–*σ*/*E*_s_ curve, resulting in inaccurate determination of the optimal energy absorption point and the best strain of CFMs. Chen et al. [20] drew the energy absorption diagram of EVA CFMs and gave the relevant empirical formulas [35].

**Figure 6:**
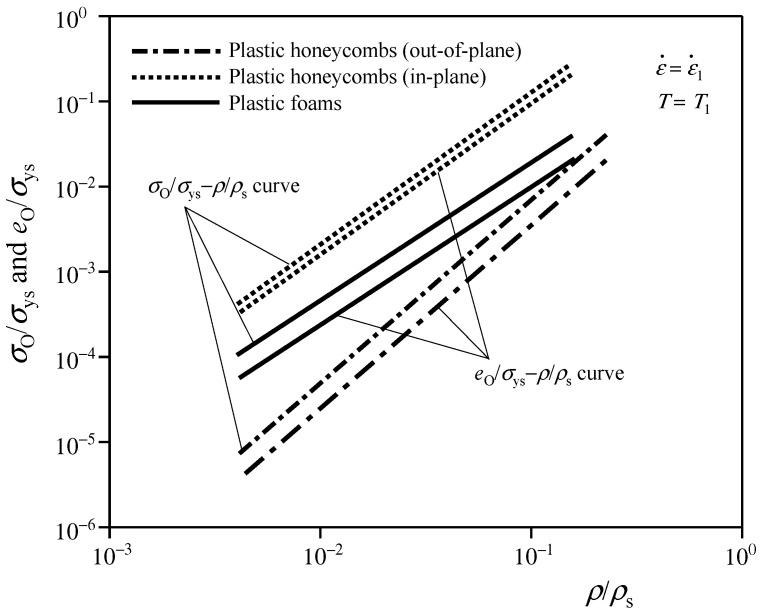
Typical *e*_O_/*σ*_ys_–*ρ*/*ρ*_s_ and *σ*_O_/*σ*_ys_–*ρ*/*ρ*_s_ curves plotted according to the method in the reference [13].

In general, the above methods cannot directly determine the final optimal density of CFMs, and there are many practical difficulties. Zhang and Ashby [13] further improved the form of energy absorption diagram, and directly established the relations between the optimal energy absorptions (optimal stresses) of CFMs normalized by the elastic moduli (yield strengths) of their base materials and their relative densities, by drawing the corresponding *e*_O_/*σ*_ys_–*ρ*/*ρ*_s_, *σ*_O_/*σ*_ys_–*ρ*/*ρ*_s_, *e*_O_/*E*_s_–*ρ*/*ρ*_s_, or *σ*_O_/*E*_s_–*ρ*/*ρ*_s_ curves, under a certain strain rate (or temperature), as shown in Figure 6. According to these curves, the density of CFM can be directly optimized.

Although the initial velocity (strain rate) at the time of impact occurrence is considered when using the energy absorption diagrams, the actual impact process with a certain mass and an initial velocity of the protected object is always accompanied by its decreasing impact velocity, which means the strain rate of CFM continuously declines. However, the energy absorption diagrams are based on the *σ*–*ε* curves of CFMs under the dynamic compressions with the initial impact velocities (strain rates) of the protected object, so the corresponding cushioning designs always overestimate the cushioning performance of CFMs, resulting in poor packages. The energy absorption diagram method is suitable for studying the influences of impact velocity, strain rate, and temperature on the energy absorption properties of CFMs, but the relevant tests still need to be carried out for the same kind of CFMs with different densities under various strain rates (or temperatures), and the test complexity and workload are still huge. Furthermore, in order to simplify the energy absorption diagram generation of CFMs, it is necessary to explore the influence of strain rate (or temperature) and density on the envelope lines in their energy absorption diagrams, which is often difficult to carry out.

To sum up, the drop hammer impact test is the most consistent with the dropping situation of a product package according to ASTM d1596-2014 [11] and GB/T 8167-2008 [19], so the constructed [*G*]–*σ*_s_ cushion curve is the standard and most widely applied in the product cushioning packaging design. Obviously, one [*G*]–*σ*_st_ cushion curve describes the relationship between [*G*] and *σ*_st_ under a certain combination of *h* and *t*. As mentioned above, to construct such cushion curves, there is a huge test workload, which prompted researchers to focus on investigating how to use the finite number of impact tests to simplify the generation process of the [*G*]–*σ*_s_ cushion curve. There are three commonly used methods—the dynamic factor method [22,23,24], dynamic stress–dynamic energy method [25,26,27,28], and dynamic cushion factor–dynamic stress method [2,29], which are detailed one by one in Section 3, Section 4 and Section 5.

## 3. Dynamic Factor Method

Sek et al. [24] proposed the dynamic factor method. The stress caused by the transient resistance of CFM to impact load can be regarded as the following two parts. One is the static stress generated due to the same deformation of the CFM under static compression *σ*_s_ (*ε*), and the other is the stress increment caused by the dynamic impact effect *σ*_d_ (*ε*,$\dot{\varepsilon }$). Therefore,

(26) $$\sigma \left(\varepsilon ,\;\dot{\varepsilon }\right)=\sigma_{s}\left(\varepsilon \right)+\sigma_{d}\left(\varepsilon ,\;\dot{\varepsilon }\right)$$

*σ*_s_ (*ε*) can be directly determined by the *σ*–*ε* curve of CFM under the quasi-static compression. Dividing all terms on both sides of the above equation by *σ*_s_ (*ε*), it can be changed as

(27) $$\frac{\sigma \left(\varepsilon ,\;\dot{\varepsilon }\right)}{\sigma_{s}\left(\varepsilon \right)}=1+\frac{\sigma_{d}\left(\varepsilon ,\;\dot{\varepsilon }\right)}{\sigma_{s}\left(\varepsilon \right)}$$

and also expressed in the simplified form of

(28) $$c\left(\varepsilon ,\;\dot{\varepsilon }\right)=1+\frac{\sigma_{d}\left(\varepsilon ,\;\dot{\varepsilon }\right)}{\sigma_{s}\left(\varepsilon \right)}$$

where the dynamic factor *c* (*ε*, $\dot{\varepsilon }$) refers to the ratio of *σ* (*ε*, $\dot{\varepsilon }$) to *σ*_s_ (*ε*). Once it is determined, in combination with the determined *σ*_s_ (*ε*), *σ* (*ε*, $\dot{\varepsilon }$) can be calculated as

(29) $$\sigma \left(\varepsilon ,\;\dot{\varepsilon }\right)=c\left(\varepsilon ,\;\dot{\varepsilon }\right)\sigma_{s}\left(\varepsilon \right)$$

In accordance with Equation (13), by using $c\left(\varepsilon ,\;\dot{\varepsilon }\right)$ and *σ*_s_ (*ε*), the *e* in the dynamic impact process is expressed as

(30) $$e=\int_{0}^{\varepsilon }c\left(\varepsilon ,\dot{\varepsilon }\right)\sigma_{s}\left(\varepsilon \right)d\varepsilon$$

For a given impact load with a known combination of a certain drop hammer mass *m*, cushioning pad thickness *t* and drop height *h*, Equation (10) is used to calculate the *σ*_st_ value. Rewriting Equation (15), the *ε*_m_ of cushioning pad is determined by

(31) $$e_{m}=\int_{0}^{\varepsilon_{m}}c\left(\varepsilon ,\dot{\varepsilon }\right)\sigma_{s}\left(\varepsilon \right)d\varepsilon =\sigma_{\mathrm{st}}\frac{h}{t}$$

If the function of max() means obtaining the maximum value of its parameter item, when *ε* ∈ (0, *ε*_m_], corresponding to Equation (17), the value of [*G*] can also be predicted by the $c\left(\varepsilon ,\;\dot{\varepsilon }\right)$ and *σ*_s_ (*ε*) as

(32) $$\left[G\right]=\frac{\max\left(\sigma \left(\varepsilon ,\dot{\varepsilon }\right)\right)}{\sigma_{\mathrm{st}}}-1=\frac{\max\left(c\left(\varepsilon ,\dot{\varepsilon }\right)\sigma_{s}\left(\varepsilon \right)\right)}{\sigma_{\mathrm{st}}}-1\begin{array}{cc} & \left(\varepsilon \in \left(0,\varepsilon_{m}\right]\right) \end{array}$$

In this way, without needing a large number of impact tests, the [*G*]–*σ*_st_ curve under a designated combination of *h* and *t* can be constructed based on the static compression *σ*–*ε* curve and $c\left(\varepsilon ,\;\dot{\varepsilon }\right)$ of the CFM. Referring to the discussion of Li et al. [22], the concrete steps are proposed as follows:

(1) For a certain combination of *h* and *t*, both *σ*–*ε* curves of cushioning pads with *t* thickness are measured under the static compression and the dynamic impact load with some total mass of drop hammer under the drop height of *h*, and then used to fit out the dynamic factor function of $c\left(\varepsilon ,\;\dot{\varepsilon }\right)$ based on proper empirical formulas.

Even though the velocity of the protected object varies during the impact process, the impact velocity (strain rate) is not high under normal product distribution conditions. The *σ*–*ε* curve of dynamic impact for some materials, such as corrugated paperboard, open-cell foam, honeycomb materials, etc., has a distinct plateau phase where the stress fluctuates slightly around a certain level. For these materials, $c\left(\varepsilon ,\;\dot{\varepsilon }\right)$ can be regarded as a constant *c*_0_, which can be calculated by using the iterative least mean squares optimization algorithm [24]. Hereby, $c\left(\varepsilon ,\;\dot{\varepsilon }\right)$ is expressed as

(33) $$c\left(\varepsilon ,\;\dot{\varepsilon }\right)=c_{0}$$

**Figure 7:**
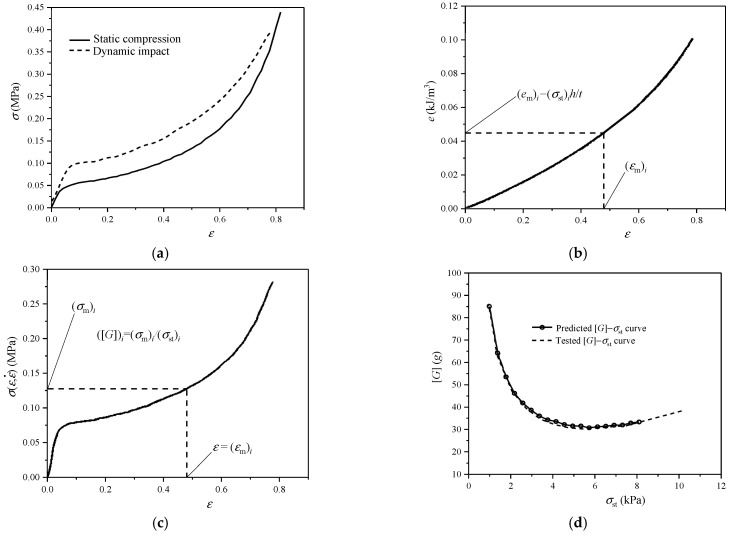
The cushion curve predicted from both *σ*–*ε* curves under static compression and dynamic impact load with a certain drop height (*h* = 60 cm) for some PE foam with a certain thickness (*t* = 50 mm) according to the method in the reference [22]: (**a**) both *σ*–*ε* curves; (**b**) predicted *e*–*ε* curve; (**c**) predicted *σ*$\left(\varepsilon ,\;\dot{\varepsilon }\right)$–*ε* curve; (**d**) [*G*]–*σ*_st_ curves.

However, $c\left(\varepsilon ,\;\dot{\varepsilon }\right)$ have a different expression for the CFMs. As shown in Figure 7a, the solid line is the static compressive *σ*–*ε* curve of some kind of CFM named polyethylene (PE) (*t* = 50 mm) with the densification deformation phase; the total mass of drop hammer is so proper that the dynamic impact energy of drop hammer just makes the cushioning pad densified, and the dotted line is its *σ*–*ε* curve of dynamic impact (*h* = 60 cm). Based on both *σ*–*ε* curves, Li et al. [22] found that $c\left(\varepsilon ,\;\dot{\varepsilon }\right)$ is linear with *ε* under a certain drop height for the CMFs, which is expressed as

(34) $$c\left(\varepsilon ,\;\dot{\varepsilon }\right)=c_{0}+I_{d}\varepsilon$$

where *I*_d_ is the growth rate of $c\left(\varepsilon ,\;\dot{\varepsilon }\right)$ on *ε*. The correlation coefficients *c*_0_ and *I*_d_ in the above equation can be calculated using the curve fitting method, based on enough data points on both *σ*–*ε* curves.

(2) Based on the determined $c\left(\varepsilon ,\;\dot{\varepsilon }\right)$ and *σ*_s_ (*ε*) of the CFM, the predicted *e*–*ε* and $\sigma \left(\varepsilon ,\dot{\varepsilon }\right)$–*ε* curves are depicted as shown in Figure 7b,c, according to Equations (30) and (29), respectively.

(3) A series of *σ*_st_ values (*σ*_st_)*_i_* of *σ*_s_(*ε*) are set, for a serial number variable *i* = 1, 2, 3… *n*_st_, where *n*_st_ is the number of *σ*_st_ values. It is assumed that the value of (*σ*_st_)*_i_* increases with the increase in *i*, and the maximum value of (*σ*_st_)*_i_* corresponds to the maximum energy absorption of the cushioning pad with thickness *t*, when it becomes densified under the impact with the drop height of *h*.

(4) Because the combination of *h* and *t* is known, for each (*σ*_st_)*_i_* value in step (3), the corresponding energy absorption per unit volume of cushioning pad (*e*_m_)*_i_*, is calculated, viz., (*e*_m_)*_i_* = (*σ*_st_)*_i_h*/*t*, according to Equation (31).

(5) For the (*e*_m_)*_i_* value calculated in step (4), the corresponding (*ε*_m_)*_i_* value is obtained from the *e*–*ε* curve (Figure 7b) plotted in step (2). During the interval of $\varepsilon \in \left(0,{\left(\varepsilon_{m}\right)}_{i}\right]$, and the corresponding maximum value of $\sigma \left(\varepsilon ,\dot{\varepsilon }\right)$, (*σ*_m_)*_i_* is acquired from the $\sigma \left(\varepsilon ,\dot{\varepsilon }\right)$–*ε* curve (Figure 7c) drawn in step (2). According to Formula (32), the corresponding [*G*] value ([*G*])*_i_* can be calculated as ([*G*])*_i_* = (*σ*_m_)*_i_*/(*σ*_st_)*_i_* − 1.

(6) For each (*σ*_st_)*_i_* value in step (3), after repeating steps (4) and (5) to obtain all of *n*_st_ points (([*G*])*_i_*, (*σ*_st_)*_i_*), the predicted [*G*]–*σ*_st_ curve can be plotted by connecting these points with the solid line, as shown in Figure 7d. It is obvious that the larger the value of *n*_st_, the smoother the constructed [*G*]–*σ*_st_ curve. The consistency between the predicted and tested [*G*]–*σ*_st_ curves verified that the proposed concrete steps of the dynamic factor method are reliable.

## 4. Dynamic Stress–Dynamic Energy Method

Burgess first proposed the dynamic stress–dynamic energy method to consolidate the [*G*]–*σ*_st_ curves of cushioning materials for different combinations of *h* and *t* [25], and two methods to generate the [*G*]–*σ*_st_ curve based on one or more than 10 drop impacts were presented [26]. For Equation (15), the left term *e*_m_ is the maximum energy absorption in the impact process, also called dynamic energy, which is a function of *ε*_m_, rewritten as

(35) $$e_{m}=\sigma_{\mathrm{st}}\frac{h}{t}=\int_{0}^{\varepsilon_{m}}\sigma \left(\varepsilon ,\dot{\varepsilon }\right)d\varepsilon =f_{1}\left(\varepsilon_{m}\right)$$

Likewise, *σ*_m_ is called dynamic stress, which is also a function of *ε*_m_, so Equation (17) can also be expressed in the form of

(36) $$\sigma_{m}=\left(\left[G\right]+1\right)\sigma_{\mathrm{st}}=f_{2}\left(\varepsilon_{m}\right)$$

Certainly, there must be a specific functional relationship between *σ*_m_ and *e*_m_ for a specific kind of CFM, that is

(37) $$\left(\left[G\right]+1\right)\sigma_{\mathrm{st}}=f\left(\sigma_{\mathrm{st}}\frac{h}{t}\right)$$

Hereby, this method is improved and applied to the generation of a cushion curve for the CFM. The concrete generation steps are established as follows:

(1) Determine the minimum *e*_mMin_ and maximum *e*_mMax_ of *e*_m_. From Equation (35), under meeting the requirements of relevant standards (ASTM D1596-2014 [11] and GB/T 8167-2008 [19]), specimen size limits, and ranges of drop hammer mass and drop height of impact testing machine, when *σ*_st_ and *h* take the minimum value, and *t* takes the maximum value, *e*_m_ = *e*_mMin_; on the contrary, *e*_m_ = *e*_mMax_, when *σ*_st_ and *h* take the maximum value, and *t* takes the minimum value. Thereupon, *e*_m_∈[*e*_mMin_, *e*_mMax_].

(2) Split the *e*_m_ from *e*_mMin_ to *e*_mMax_ into *n*_e_ values with approximately equal intervals. *n*_e_ is the number of *e*_m_ values, and generally *n*_e_ = 10.

(3) For each *e*_m_ value *e*_m*i*_ in step (2) (*i* = 1, 2, 3... 10), take five different combinations of (*σ*_st_)*_j_*, (*h*)*_j_* and (*t*)*_j_* (hereby another serial number variable *j* = 1, 2, 3... 5), all of which satisfy that (*σ*_st_)*_j_*(*h*)*_j_*/(*t*)*_j_* = *e*_m*i*_. The tested specimens have the uniform bearing area *A* in the impact direction. According to *A* and (*σ*_st_)*_j_*, the corresponding drop hammer mass (*m*)*_j_* is calculated using Equation (10). Under each combination of (*m*)*_j_*, (*h*)*_j_*, and (*t*)*_j_*, the corresponding impact test is carried out according with the relevant standards (ASTM D1596-2014 [11] and GB/T 8167-2008 [19]) to obtain the corresponding [*G*] value ([*G*])*_j_*, so as to calculate the corresponding dynamic stress (*σ*_m_)*_j_* using Equation (36). According to the above theory, for the same kind of CFM, under these five different combinations, all tested specimens have the same dynamic stress *σ*_m*i*_ corresponding to *e*_m*i*_. That is to say, *σ*_m*i*_ =$\sum_{j=1}^{5}{\left(\sigma_{m}\right)}_{j}/5$.

(4) For each *e*_m*i*_ value in step (2), step (3) is repeated, so as to obtain all *n*_e_ value points of dynamic stress and dynamic energy, (*σ*_m*i*_, *e*_m*i*_), *i* = 1, 2, 3... *n*_e_.

(5) Derive the empirical relationship expression between dynamic stress and dynamic energy by fitting the *n*_e_ points of (*σ*_m*i*_, *e*_m*i*_) obtained in step (4) based on a certain relationship equation. For most CFMs, their cushioning mechanism relies on air compression. Like the materials of corrugated paper fiberboard, honeycomb paperboard, bubble pad, and air column pillow, the primary cushioning principle of CFMs is related to the behavior of the air entrapped within their closed cells, relying on its displacement. In contrast, the cushioning mechanism of open-cell foam materials (such as expanded polyurethane) relies on mechanical means for their cushioning performance, which will probably require a different model. Based on the ideal gas model theory, when the dynamic stress and dynamic energy of CFMs have the same units, the following relationship is satisfied numerically as [28]

(38) $$\left(\left[G\right]+1\right)\sigma_{\mathrm{st}}=ae^{b\left(\sigma_{\mathrm{st}}\frac{h}{t}\right)}$$

where *a* and *b* are dimensionless relationship coefficients, and their values depend on the kinds and densities of CFMs; e is the natural constant; and e = 2.71828.

(6) Use the empirical relationship expression between dynamic stress and dynamic energy to generate the [*G*]–*σ*_st_ cushion curves. The minimum and maximum values are assumed as (*σ*_st_)_Min_ and (*σ*_st_)_Max_, respectively. For a certain combination of *h* and *t*, corresponding to each value of *σ*_st_ in the range of [(*σ*_st_)_Min_, (*σ*_st_)_Max_], the [*G*] value is calculated from the empirical relationship expression, such as the expression (38).

**Figure 8:**
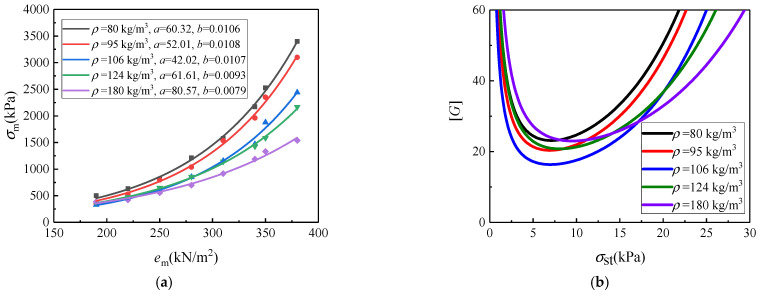
Constructing the cushion curves using the dynamic stress-dynamic energy method for some EVA CFMs with different densities and a certain thickness (*t* = 30 mm) under a certain drop height (*h* = 40 cm): (**a**) the fitted *σ*_m_–*e*_m_ curves; (**b**) the predicted [*G*]–*σ*_st_ curves.

For some kind of EVA CFMs with different densities, the relationship curves between their dynamic stress and dynamic energy (*n*_e_ = 8) are depicted using the above steps from (1) to (5), as shown in Figure 8a. Under the combination of *h* = 40 cm and *t* = 30 mm, the constructed [*G*]–*σ*_st_ cushion curves are depicted in Figure 8b, for these EVA CFMs. For the CFMs, the cushioning mechanism mainly relies on the behavior of the air entrapped within their closed cells, besides the mechanical means of their cell edges and faces. For the CFMs with the same base material, as the density increases, the proportion of the solid base material (cell edges and cell faces) increases, and their contribution to cushioning performance becomes larger. Although the contribution of the entrapped air still dominates, meaning the formula (38) still satisfies, its contribution weakens accordingly. The combined effect of both causes the dependence of the cushioning curves on the increasing densities of CFMs in a non-additive manner (Figure 8b).

## 5. Dynamic Cushion Factor–Dynamic Energy Method

For one [*G*]–*σ*_st_ curve with a given combination of *h* and *t*, the *C* and *e*_m_ values are calculated according to Equations (20) and (35), and both are variables on *σ*_st_, hereby rewritten as

(39) $$C=\frac{\sigma_{m}}{e_{m}}=\frac{\left[G\right]+1}{h/t}=f_{3}\left(\varepsilon_{m}\right)$$

and

(40) $$e_{m}=\sigma_{\mathrm{st}}\frac{h}{t}=f_{1}\left(\varepsilon_{m}\right)$$

The corresponding *C*–*e*_m_ curve can be obtained from one [*G*]–*σ*_st_ curve according to the above two equations. In fact, each coordinate point on the *C*–*e*_m_ curve can correspond to a series of ([*G*], *σ*_st_) point sets on multiple [*G*]–*σ*_st_ cushion curves with different combinations of *h* and *t* for the same kind of CFM, which makes the *C*–*e*_m_ curve no longer depend on the specific combination of *h* and *t* anymore and become more general. In essence, like *e*_m_, *C* is also called the dynamic cushion factor and is also a function of *ε*_m_. Therefore, for a kind of CFM, there must be a certain functional relationship between *C* and *e*_m_. Based on the above two equations, Ge [29] proposed one kind of simplified construction method of [*G*]–*σ*_st_ cushion curve. The concrete steps are established as follows:

(1) Like the first step of dynamic stress–dynamic energy method, determine the minimum *e*_mMin_ and maximum *e*_mMax_ of *e*_m_. Likewise, under meeting the relevant test standards, specimen size requirements, and ranges of drop hammer mass and drop height of impact test machine, when *σ*_st_ = (*σ*_st_)_Min_ and *h* takes the minimum value, and *t* takes the maximum value, *e*_m_ = *e*_mMin_; on the contrary, *e*_m_ = *e*_mMax_. Likewise, *e*_m_ ∈ [*e*_mMin_, *e*_mMax_].

(2) As shown in Table 1, the first and second columns are *σ*_st_ and *h*/*t* ratio of some kind of CFMs under dynamic impacts, respectively, and the corresponding *e*_m_ value is calculated according to Equation (40), as listed in the third column. It should be notable that the *e*_m_ value increases from *e*_mMin_ to *e*_mMax_ by increasing both values of *σ*_st_ and *h*/*t* ratio, increasing only one with the other fixed, increasing both alternately, or randomly assigning values for both, as the number of rows increases. Supposing that the interval of *e*_m_ is divided into *n*_c_ values of *e*_m_, each of which is *e*_m*i*_, and for the convenience of demonstration, *n*_c_ = 6 (*i* = 1, 2, 3... *n*_c_), as in column 3 of Table 1.

(3) Corresponding to each line of Table 1, for each *e*_m*i*_ value in step (2), in order to ensure accuracy, five different combinations of *h* and *t* are taken, while ensuring that the values of *σ*_st_ and *h*/*t* ratio are constant. According to the relevant test standards, all specimens have the same cross-sectional area *A*, and the *m* values can be determined for the five combinations. The corresponding impact tests are carried out to obtain the tested [*G*] values. Additionally, for the five different combinations, all tested specimens with the same kind of CFM should have the same [*G*] value [*G*]*_i_*. The average of tested [*G*] values for five combinations is [*G*]*_i_* corresponding to *e*_m*i*_, as listed in column 4 of Table 1. Using Equation (39), the corresponding *C* value can be calculated, as listed in column 5 of Table 1. When *n*_c_ is sufficiently large, the *C*–*e*_m_ curve of the CFM is determined by means of curve fitting, based on *n*_c_ value points.

(4) Corresponding to the value points on the *C*–*e*_m_ curve, for certain *h*/*t* ratios, the [*G*] and *σ*_st_ values are calculated using Equations (39) and (40), respectively, to extrapolate the final [*G*]–*σ*_st_ curves, as shown in Table 2.

## 6. Dynamic Cushion Factor–Dynamic Stress Method

As shown above, *σ*_m_, *e*_m_, and *C* are all functions of *ε*_m_. For a certain kind of CFM, there are definite functional relationships between *σ*_m_ and *e*_m_, and *C* and *e*_m_, so there must be a definite functional relationship between *C* and *σ*_m_. Their expressions are presented again as follows:

(41) $$C=\frac{\left[G\right]+1}{h/t}=f_{3}\left(\varepsilon_{m}\right)$$

(42) $$\sigma_{m}=\left(\left[G\right]+1\right)\sigma_{\mathrm{st}}=f_{2}\left(\varepsilon_{m}\right)$$

Referring to the dynamic cushion factor–dynamic energy method, the construction steps of the dynamic cushion factor–dynamic stress method are proposed as follows:

(1) Like the first two steps of dynamic cushion factor–dynamic energy method, under meeting the relevant test standards, specimen size requirements, and ranges of drop hammer mass and drop height of impact test machine, and guaranteeing that the *e*_m_ value increases from *e*_mMin_ to *e*_mMax_ by increasing both values of *σ*_st_ and *h*/*t* ratio, increasing only one with another fixed, increasing both alternately, or randomly assigning values for both, as the row number increases, the values of *σ*_st_ and *h*/*t* ratio are listed in the first two columns of Table 3, respectively. For the convenience of explanation, the values of *σ*_st_ and *h*/*t* ratio used in the dynamic cushion factor–dynamic energy method are directly taken, and the number of value points on the cushion curves *n*_m_ = 6 (*i* = 1, 2, 3... *n*_m_) too.

(2) Likewise, for a kind of CFM, when the values of *σ*_st_ and *h*/*t* ratio are fixed, there is a certain value of [*G*]. Corresponding to a certain combination of *σ*_st_ and *h*/*t* ratio, five group combinations with different values of *h*, *t*, and *m* are determined, and the relevant hammer impact tests can be carried out to obtain the tested values of [*G*]. The average [*G*] values are listed in column 3 of Table 3. According to Equations (41) and (42), the corresponding values of *C* (column 4 of Table 3) and *σ*_m_ (column 5 of Table 3) are calculated. The *C*–*σ*_m_ curve of the CFM is also determined by means of curve fitting.

(3) Corresponding to the value points on the *C*–*σ*_m_ curve, for certain *h*/*t* ratios, the [*G*] and *σ*_st_ values are calculated using Equations (41) and (42), respectively, to extrapolate the final [*G*]–*σ*_st_ curves, as listed in Table 4.

## 7. Conclusions

This paper reviews the development history of constructing cushion curves, which mainly includes the methods of Janssen factor, Rusch curve, cushion factor, and energy absorption diagram. The Janssen factor can be used to evaluate the acceleration efficiencies about energy absorption for different CFMs, but it fails to link the energy absorption with the deformation mechanism of CFM; it is only an empirical measurement and needs to collect a large number of test data by carrying out the impact tests for the cushioning pads with different thicknesses and densities. The Rusch curve method is more general than the Janssen factor method, relying on the empirical function describing the stress–strain curve of CFM, but lacks any mechanical principle. The cushioning theory is systematically summarized, and three parameters describing the cushioning performance of CFM are derived: dynamic cushion factor, dynamic stress, and dynamic energy. Based on this theory and the hammer impact test method, the maximum acceleration–static stress curve of CFM with a certain thickness can be obtained under a certain drop height. Using the dynamic compression test method, the cushion factor–stress curve of CFM can also be obtained; during the whole dynamic compression process, the compressive velocity (strain rate) remains constant, which is different from the continuous decline of the impact velocity in the actual package dropping process, and therefore, this is bound to overestimate the cushioning performance of materials, resulting in poor packaging; in order to optimize the CFM and its density, it is necessary to carry out a large number of dynamic compression experiments for different CFMs with different densities, which becomes complex and even impractical. The energy absorption diagram is a set of envelope lines of the normalized energy absorption per unit volume–stress curves obtained from the compression stress–strain curves of the CFMs with different densities under various strain rates (or temperatures). The energy absorption diagram is obtained based on the dynamic compression of CFM, while the actual product drop is an impact process with a certain mass and initial speed, in which the velocity of the protected object first decreases continuously, so the cushioning design based on this method also overestimates the cushioning performance of materials, resulting in poor packaging.

The hammer impact test loading is the most consistent with the dropping situation of the product package, and it is the most standard and widely applied to use the maximum acceleration–static stress curves based on the hammer impact tests for realizing the cushioning packaging design. However, in order to construct such cushion curves, a large number of impact tests should be carried out for the CFMs with different thicknesses by changing the weight of the drop hammer under different drop heights, and the test workload is extremely large. The maximum acceleration–static curves of CFMs can be predicted by means of finite impact tests, by using the dynamic factor, dynamic stress-dynamic energy, and dynamic cushion factor-dynamic energy methods. In Section 3, Section 4 and Section 5, the construction principles and concrete generation steps of cushion curves are presented in detail. These three methods significantly reduce the number of tests and improve the generation efficiency of cushion curves. Inspired by these methods, the novel dynamic cushion coefficient-dynamic stress method is proposed. For this method, the static stress and *h*/*t* ratio firstly take different values to ensure the increase in dynamic energy; for each certain combination of *σ*_st_ and *h*/*t* ratio, the hammer impact tests with five different combinations of *m*, *h* and *t* are carried out to obtain the average of measured [*G*] values, and the corresponding values of *C* and *σ*_m_ are calculated, so as to obtain the fitted *C*–*σ*_m_ curve; according to the obtained *C*–*σ*_m_ curve, for a specific *h*/*t* ratio, the corresponding [*G*] and *σ*_st_ values can be predicted to construct the final [*G*]–*σ*_st_ cushion curve.

Based on the above practical generation steps of constructing cushion curves through four simplified methods, the specific software for cushion curve generation and cushioning design of CFMs can be developed by using the related computer programming technology, which will better promote the rational use of CFMs and the conservation of relative material resources.

## Figures and Tables

**Table 1:** Determination of coordinate points on the *C*–*e*_m_ curve corresponding to steps (1)–(3) of dynamic cushion factor-dynamic energy method.

*σ*_st_ (kN/m^2^)	*h*/*t*	*e*_m_ (kN/m^2^)	[*G*]	*C*
27.5959	6	165.5755	41.7102	7.1184
42.1980	6	253.1881	24.8105	4.3018
45.6107	9	410.4966	27.0012	3.1112
38.4028	19	729.6540	50.1002	2.6895
42.1980	21	886.1583	59.1037	2.8621
45.6107	21	957.8253	82.6125	3.9815

**Table 2:** The [*G*]–*σ*_st_ curves are extrapolated from the *C*–*e*_m_ curve of some CFM.

*C*	*e*_m_ (kN/m^2^)	*h*/*t*									
		6		9		14		19		21	
		*σ*_st_ (kN/m^2^)	[*G*]	*σ*_st_ (kN/m^2^)	[*G*]	*σ*_st_ (kN/m^2^)	[*G*]	*σ*_st_ (kN/m^2^)	[*G*]	*σ*_st_ (kN/m^2^)	[*G*]
7.1184	165.5755	27.5959	41.7102	18.3973	63.0653	11.8268	98.6571	8.7145	134.2490	7.8845	148.4857
4.3018	253.1881	42.1980	24.8105	28.1320	37.7158	18.0849	59.2245	13.3257	80.7333	12.0566	89.3368
3.1112	410.4966	68.4161	17.6675	45.6107	27.0012	29.3212	42.5574	21.6051	58.1136	19.5475	64.3361
2.6895	729.6540	121.6090	15.1369	81.0727	23.2054	52.1181	36.6528	38.4028	50.1002	34.7454	55.4792
2.8621	886.1583	147.6931	16.1725	98.4620	24.7587	63.2970	39.0691	46.6399	53.3795	42.1980	59.1037
3.9815	957.8253	159.6376	22.8893	106.4250	34.8339	68.4161	54.7417	50.4119	74.6494	45.6107	82.6125

**Table 3:** Determination of coordinate points on the *C*–*σ*_m_ curve corresponding to steps (1) and (2) of dynamic cushion factor–dynamic stress method.

*σ*_st_ (kN/m^2^)	*h*/*t*	[*G*]	*C*	*σ*_m_ (kN/m^2^)
27.5959	6	41.7102	7.1184	1178.6270
42.1980	6	24.8105	4.3018	1089.1519
45.6107	9	27.0012	3.1112	1277.1551
38.4028	19	50.1002	2.6895	1962.3929
42.1980	21	59.1037	2.8621	2536.2568
45.6107	21	82.6125	3.9815	3813.6271

**Table 4:** The [*G*]–*σ*_st_ curves are extrapolated from the *C*–*σ*_m_ curve of some CFM.

*C*	*σ*_m_ (kN/m^2^)	*h*/*t*									
		6		9		14		19		21	
		*σ*_st_ (kN/m^2^)	[*G*]	*σ*_st_ (kN/m^2^)	[*G*]	*σ*_st_ (kN/m^2^)	[*G*]	*σ*_st_ (kN/m^2^)	[*G*]	*σ*_st_ (kN/m^2^)	[*G*]
7.1184	1178.6270	27.5959	41.7102	18.3973	63.0653	11.8268	98.6571	8.7145	134.2490	7.8845	148.4857
4.3018	1089.1519	42.1980	24.8105	28.1320	37.7158	18.0849	59.2245	13.3257	80.7333	12.0566	89.3368
3.1112	1277.1551	68.4161	17.6675	45.6107	27.0012	29.3212	42.5574	21.6051	58.1136	19.5475	64.3361
2.6895	1962.3929	121.6090	15.1369	81.0727	23.2054	52.1181	36.6528	38.4028	50.1002	34.7454	55.4792
2.8621	2536.2568	147.6931	16.1725	98.4620	24.7587	63.2970	39.0691	46.6399	53.3795	42.1980	59.1037
3.9815	3813.6271	159.6376	22.8893	106.4250	34.8339	68.4161	54.7417	50.4119	74.6494	45.6107	82.6125

## Data Availability

Data are contained within the article.

## Abbreviations

The following abbreviations are used in this manuscript:

CFM	Closed-cell foam material
EVA	Closed-cell ethylene-vinyl acetate
PE	Polyethylene
